# Dimensional Fidelity and Slicer Mass Prediction Bias in FFF-Printed UAV Micro-Frames: A Material-Dependent Comparative Study

**DOI:** 10.3390/ma19081507

**Published:** 2026-04-09

**Authors:** Panagiotis Panagos, Antreas Kantaros, Theodore Ganetsos, Michail Papoutsidakis

**Affiliations:** Department of Industrial Design and Production Engineering, University of West Attica, 12244 Athens, Greece; mscdrones8096672@uniwa.gr (P.P.); ganetsos@uniwa.gr (T.G.); mipapou@uniwa.gr (M.P.)

**Keywords:** 3D printing, additive manufacturing, fused filament fabrication, dimensional accuracy, UAV

## Abstract

**Highlights:**

- ABS prints exhibited pronounced warping and dimensional instability.
- PLA and PETG mainly showed surface-related defects without macroscopic warping.
- PLA achieved the highest dimensional consistency across the tested configurations.
- Slicer mass prediction showed strong material-dependent bias.
- Infill reduction had limited influence on part mass at the micro-frame scale.

**Abstract:**

**Objective:** This study investigates the influence of selecting three thermoplastics as raw materials (PLA, PETG, and ABS) on dimensional accuracy, defect formation, and slicer-based mass prediction reliability in FFF 3D-printed UAV micro-frames. **Methods:** A factorial experimental design combining three materials, two micro-frame geometries, and two infill levels was implemented. Print quality was assessed through structured visual inspection of common FFF defects, while manufacturing reliability was evaluated by comparing slicer-predicted and experimentally measured mass. Dimensional fidelity was quantified at critical motor mount features using repeated micrometric measurements and dedicated accuracy and uniformity indices. **Results:** The results reveal strong material-dependent behaviour. PLA exhibited the highest dimensional consistency and near-zero mean mass prediction error, PETG showed intermediate performance, and ABS presented significant warping, together with a pronounced positive mass prediction bias. These findings indicate systematic discrepancies between predicted and measured mass values and highlight the need for material-dependent calibration of slicing software. **Conclusions:** Material selection and process calibration strongly affect dimensional fidelity and manufacturing reliability in FFF-printed UAV micro-frames. The findings provide practical guidance for material choice and slicing parameter adjustment in UAV fabrication and similar small-scale FFF applications.

## 1. Introduction

Micro-frames of Unmanned Aerial Vehicles (UAVs) represent lightweight polymer structures subject to strict constraints in mass, geometry, and assembly tolerances. At this scale, even sub-millimetre deviations on functional surfaces, including motor mounts and fastening holes, can degrade assembly quality, disturb thrust alignment, and reduce operational reliability. Within the broader context of additive manufacturing (AM) and according to standardised terminology, Material Extrusion (MEX), particularly fused filament fabrication (FFF), historically known as Fused Deposition Modelling (FDM), has become an accessible approach for producing low-cost polymer components with rapid design iteration capability [1,2]. Its significance is reinforced by the direct transition from a digital model to a physical prototype, which enables rapid design iteration and manufacturing flexibility in lightweight polymer structures [3].

In FFF, a thermoplastic filament is melted and deposited layer by layer, and the quality of the final part depends on both intrinsic material behaviour and process settings [4,5,6]. Key factors include interlayer bonding, which is governed by diffusion and melt welding and is associated with anisotropy, as well as thermal effects such as shrinkage and residual stresses that may lead to dimensional deviations and warping, particularly in materials such as ABS [7,8]. In addition, surface defects such as stringing, zits, and visible layer lines are commonly observed, especially in geometries with small features.

Despite these challenges, FFF offers practical advantages in novel UAV development. It enables on-demand part production, rapid design modification, and component replacement with minimal tooling cost. The relevance of FFF to UAV applications has been established in the literature, both in terms of manufacturing feasibility and the functional utilisation of polymer components [9,10]. Reviews focusing on drone parts highlight growing interest in advanced filaments, including fibre-reinforced composites, to improve the stiffness-to-weight ratio and reduce mass [11]. Recent discussion on high-speed thermoplastics and benchtop AM ecosystems emphasises that print quality depends on the combined selection of material, software, and operational strategy [12,13]. At the material level, comparative studies indicate that PLA, ABS, and PETG do not share an equivalent sensitivity to process parameters, resulting in distinct process windows [14]. For PETG, both thermochemical and mechanical comparisons with PLA have been reported [15], together with parameter optimisation studies that target more stable mechanical performance [16].

Nevertheless, a substantial portion of prior work evaluates quality primarily through standardised specimens and strength-related metrics, with limited emphasis on complex lightweight geometries such as UAV micro-frames. In particular, there is limited systematic evidence on how commonly used thermoplastics influence dimensional fidelity and slicer mass prediction accuracy in complex, small-scale geometries that incorporate functional assembly features. In such parts, feature density, thin walls, and small radii make dimensional consistency, surface integrity, and mass estimation equally critical. Furthermore, although infill density and infill pattern are known to affect printed parts’ responses [17], the behaviour of honeycomb-type structures and their interaction with process settings are not always quantified for applications that include functional assembly features [18,19]. Multi-objective approaches that jointly weigh print time and shape accuracy highlight that optimal settings typically represent a compromise [20], while broader reviews confirm the multifactorial nature of parameter selection when overall part quality is the objective [21,22,23,24,25,26,27,28,29]. For such geometries, manufacturing fidelity—expressed through dimensional accuracy, defect manifestation, and process predictability—becomes a critical performance dimension alongside, and sometimes prior to, mechanical characterisation.

On this basis, the present study investigates the influence of material selection on the fabrication of UAV micro-frames using fused filament fabrication. A factorial experimental design is employed, combining three thermoplastic materials (ABS, PETG, and PLA), two representative micro-frame geometries (micro105 and MK8), and two infill levels (35% and 100%), using a common slicing workflow. The analysis focuses on manufacturing fidelity and process reliability, including defect manifestation, slicer mass prediction accuracy, and dimensional consistency at functionally critical motor mount features. The study contributes a systematic, application-oriented comparison of material-dependent manufacturing fidelity in FFF-printed UAV micro-frames, integrating defect assessment, dimensional metrology, and slicer mass prediction analysis within a unified experimental framework. Within this framework, the study examines whether material selection leads to observable differences in dimensional fidelity and slicer mass prediction behaviour under comparable processing conditions. Accordingly, the null hypothesis of the study is defined as follows. **H_0_:** There is no significant difference between PLA, PETG, and ABS in terms of dimensional fidelity or slicer mass prediction behaviour under the examined FFF processing conditions.

## 2. Materials and Methods

### 2.1. Experimental Design

Twelve UAV micro-frames were fabricated by FFF using a full-factorial 3 × 2 × 2 experimental design with three materials, ABS, PETG, and PLA, two frame geometries, micro105 and MK8, and two infill levels, 35% and 100%. The 35% condition used a honeycomb infill architecture, while the 100% condition produced a fully solid interior.

The CAD models were obtained from publicly available repositories. The micro105 design was obtained from a Thingiverse listing titled “Micro 105 FPV Quadcopter” (https://www.thingiverse.com/thing:1221911, accessed on 12 November 2025). The MK8 design was obtained from MyMiniFactory, a listing titled “MK XIII Micro Quad” (https://www.myminifactory.com/object/3d-print-mk-xiii-micro-quad-28907, accessed on 14 November 2025). The downloaded STL files were used without geometric modification, except that only the main plate of the micro105 design was printed. Each factor combination was printed once, yielding 12 unique specimens. The full-factorial design allows the principal effects of material, geometry, and infill level to be examined within a controlled experimental space while maintaining a manageable fabrication effort.

The use of openly available designs reflects the workflow commonly followed by hobbyists, researchers, and small UAV developers, where open-source repositories serve as a primary source of printable drone components. This choice therefore enables the experimental evaluation to reflect realistic additive manufacturing practices in the UAV community.

### 2.2. Materials, Hardware, and Slicing Workflow

Commercial eSUN filaments with a nominal diameter of 1.75 mm were employed. Manufacturer-declared nominal densities were used when density input was required for slicer mass prediction. Three laboratory printers were used, one per material, based on system availability. PLA specimens were fabricated on a Creality CR-20 (Creality, Shenzhen, China) open-frame printer, PETG specimens on an Anycubic i3 Mega (Anycubic, Shenzhen, China) open-frame printer, and ABS specimens on an enclosed Anycubic 4Max Pro (Anycubic, Shenzhen, China) printer, selected to ensure a more tightly controlled printing environment. All models were prepared in Simplify3D v4.1.2. The use of different printers reflects a typical laboratory workflow in which material selection is often coupled with printer configuration and environmental control (e.g., enclosed vs. open-frame systems). It is acknowledged that this introduces additional process-dependent factors, such as thermal stability and machine-specific calibration, which are not fully decoupled from intrinsic material behaviour. Accordingly, the present results should be interpreted as reflecting the combined influence of material and process environment within a realistic benchtop additive manufacturing context.

While detailed molecular characteristics such as molecular weight are not typically disclosed by filament manufacturers, differences in melt flow behaviour between the examined materials are implicitly reflected in their processing response during extrusion. These differences, which are closely related to melt viscosity and flow stability, are considered in the interpretation of the results presented in Section 4.

To ensure comparability across specimens, the core process parameters were kept common across all builds, as summarised in Table 1. A 0.4 mm nozzle was used throughout, the extrusion width was set to 100%, and the print speed was set to 45 mm/s. Material-dependent setpoints comprised nozzle temperature, bed temperature, extrusion multiplier, and retraction settings, as listed in Table 1. First-layer settings were adjusted to improve adhesion and reduce base distortion by setting the first-layer height to 90% of the nominal layer height and reducing first-layer print speed to 36 mm/s for PLA and PETG and to 22.5 mm/s for ABS. The raster angle during deposition followed the default slicing strategy of the employed software, consisting of alternating raster orientations between successive layers (typically 0°/90°), ensuring balanced in-plane material distribution. For the 35% infill condition, a honeycomb pattern was used, while the 100% condition was printed fully solid.

Because ABS is prone to warping, a bed-adhesion aid was selectively used in two ABS builds to increase adhesion and mitigate edge lift. This choice reflects common FFF practice for ABS processing and was applied in a controlled manner to stabilise builds where warping risk was expected to be highest. A commercially available glue was applied between the part and the heated build surface for micro105 at 100% infill and for MK8 at 35% infill. The remaining ABS builds were printed without glue.

### 2.3. Post-Processing and Qualitative Defect Assessment

Macroscopic print defects were documented on the as-printed specimens prior to post-processing. Representative photographs were recorded both before and after post-processing. Post-processing was intentionally limited to the removal of superficial artefacts rather than cosmetic finishing. Stringing was removed using small scissors, and zits were reduced using a fine baby nail file to minimise collateral surface damage.

Print defects were recorded visually using a structured qualitative scale for four defect types, stringing, zits, layer lines, and warping. Defect intensity was categorised as none, slight, moderate, or pronounced. The assessment was intended as a comparative descriptor within the present dataset rather than a universal defect taxonomy, allowing consistent comparison between materials under identical printing conditions.

### 2.4. Mass Measurement and Slicer Mass Estimation Error

All mass measurements were performed after post-processing. Specimen mass was measured using a Tele CX-Series digital balance with 0.01 g resolution. Before measurements, the balance was calibrated using a 50 g reference mass to ensure measurement consistency. Weighing was conducted on a stable surface away from drafts and vibrations.

For each specimen, the real mass was computed as the arithmetic mean of five consecutive measurements and was reported with two decimals, consistent with instrument resolution. A conservative measurement uncertainty of ±0.01 g, equal to the balance resolution, was adopted for all values. This choice was supported by replicate weighing standard deviations of 0.004–0.009 g, which are below the instrument resolution. Using an uncertainty of the mean would yield values below 0.01 g and would not capture additional contributions such as potential calibration bias. All measurements were performed under consistent environmental and procedural conditions to minimise variability.

The slicer-estimated mass m_slicer_ was taken directly from Simplify3D output. Mass prediction error was quantified as a relative percentage, as given in Equation (1):

(1) $$\varepsilon \;\left(\%\right)\;=\left(\frac{m_{\mathrm{slicer}}\;-{\;m}_{\mathrm{real}}}{m_{\mathrm{real}}}\right)\times \;100$$

At the group level, *ε* was summarised by material (n = 4 per material) and by design (n = 6 per design) using the mean and sample standard deviation to describe systematic bias and dispersion. In addition, the overall mean percentage error across all twelve specimens was computed as ${\bar{\varepsilon }}_{\mathrm{all}}$ and used as the reference mean in the per-specimen and grouped visualisations.

Agreement between m_slicer_ and m_real_ was further examined using the Bland–Altman approach on a percentage basis. The x-axis represented the mean of the two methods, (m_slicer_ + m_real_)/2, and the y-axis represented *ε*. Overall dispersion across all specimens was quantified as s*_ε_*_,all_, and the 95% limits of agreement (LoAs) were defined as ${\bar{\varepsilon }}_{\mathrm{all}}$ ± 1.96 × s*_ε_*_,all_.

### 2.5. Dimensional Metrology and Indices

Dimensional performance was evaluated at a functionally critical feature, the height of the four motor mount bases on each frame. Measurements were taken using an Ingco HDCD28150 digital micrometre (Ingco, Suzhou, Jiangsu, China) with 0.01 mm accuracy. Dimensional measurements were performed after post-processing and after mass measurements, using the same specimens.

For each specimen, each motor mount base was measured five times using a standardised procedure to minimise operator influence and ensure measurement consistency. The micrometre was re-zeroed before moving to the next base to maintain measurement accuracy throughout the process. Bases were indexed κ = 1, 2, 3, 4. Nominal motor mount heights were taken from the CAD models: h_nom_ = 7.50 mm for micro105 and h_nom_ = 10.00 mm for MK8.

For each base κ, the mean height ${\bar{h}}_{k}$ and sample standard deviation s_κ_ were computed from the five repeated measurements. The absolute deviation from nominal was defined as ∣Δh_k_∣ = ∣${\bar{h}}_{k}$ − h_nom_∣. Two frame-level indices were then calculated. The accuracy index was defined as the mean absolute deviation across the four bases:

(2) $$A_{\mathrm{UAV}}\;=\;\frac{1}{4}\overset{4}{\underset{k=1}{\sum }}|{\Delta h}_{k}|\;$$

The uniformity index was defined as the sample’s standard deviation of the four mean base heights:

(3) $$U_{\mathrm{UAV}}\;=\sqrt{\frac{1}{3}\overset{4}{\underset{k=1}{\sum }}{({\bar{h}}_{k}\;-\;\mu )}^{2}}$$

where μ is defined as follows:

(4) $$\mu \;=\;\frac{1}{4}\overset{4}{\underset{k=1}{\sum }}{\bar{h}}_{k}$$

To enable a material-centric comparison of dimensional performance, two aggregated indices were defined for each material: A_material_ and U_material_. For a given material, A_material_ was computed as the arithmetic mean of the four A_UAV_ values obtained across the combinations of geometry and infill (two designs and two infill levels):

(5) $$A_{\mathrm{material}}\;=\;\frac{1}{4}\overset{4}{\underset{i=1}{\sum }}A_{\mathrm{UAV},i}$$

Analogously, U_material_ was computed as the arithmetic mean of the corresponding four U_UAV_ values:

(6) $$U_{\mathrm{material}}\;=\;\frac{1}{4}\overset{4}{\underset{i=1}{\sum }}U_{\mathrm{UAV},i}$$

These material-level indices provide a compact summary of typical deviation from nominal motor mount height and typical inter-base uniformity, while averaging out geometry and infill effects within the studied design space. They were used for the cross-material comparative visualisation reported in the Results.

## 3. Results

The results are presented in three structured parts. First, macroscopic print defects are documented through qualitative inspection and summarised in Table 2. Second, slicer mass prediction error is quantified at both specimen and grouped levels (Table 3 and Table 4, Figures 3–5). Third, dimensional consistency is evaluated through defined indices and summarised at both specimen and material levels (Table 5, Table 6 and Table 7, Figure 7). First, macroscopic print defects are documented through structured visual inspection and qualitative grading. Second, slicer mass prediction is quantified using the percentage error *ε* between m_slicer_ and mr_eal_, reported at specimen level and after grouping by material and by frame design. Third, dimensional consistency is evaluated at the motor mount bases using the indices A_UAV_ and U_UAV_, and summarised at material level through A_material_ and U_material_. These results are discussed primarily in terms of process–material interactions affecting dimensional fidelity and manufacturing reliability.

### 3.1. Print Quality and Defects

**Figure 1:**
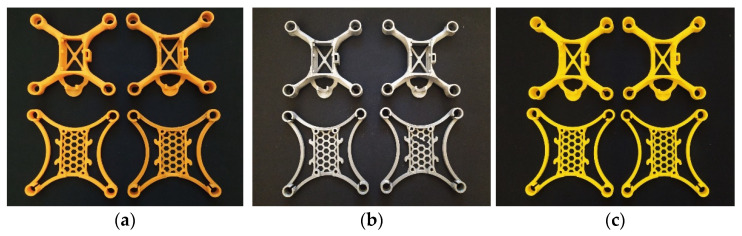
Printed UAV micro-frames after post-processing: (**a**) ABS, (**b**) PETG and (**c**) PLA.

Figure 1 presents representative printed UAV micro-frames after post-processing for each material.

A concise qualitative grading of print defects is provided in Table 2, based on the as-printed inspection conducted prior to post-processing. The grading captures surface artefacts, including stringing, zits, layer lines and gross deformation, namely warping.

**Figure 2:**
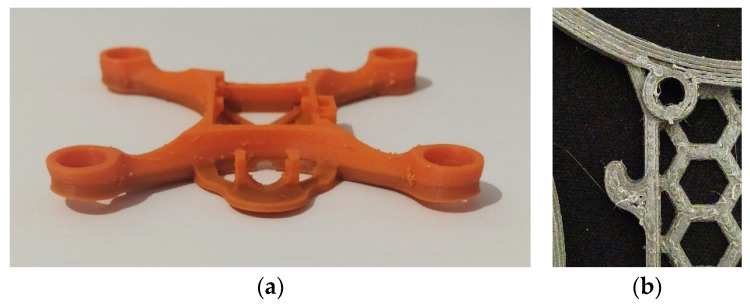
Representative defects prior to post-processing: (**a**) warping and zits in an ABS MK8 micro-frame printed at 100% infill; (**b**) layer lines and stringing in a PETG micro105 micro-frame printed at 35% infill.

Across the present dataset, ABS exhibited the most severe macroscopic defects. Warping, observed as edge lift, co-occurred with pronounced zits and visible degradation of geometric integrity. This behaviour is illustrated in Figure 2a and is consistent with the qualitative grading reported for ABS in Table 2.

In contrast, PLA and PETG did not exhibit macroscopic warping in the inspected specimens. Their dominant defects were surface-related and appeared as combinations of stringing, zits, and layer lines. Qualitative grading indicates that defect manifestation was not purely material driven. For PETG, layer lines were more pronounced in MK8 than in micro105, indicating that geometry and toolpath interactions can visibly affect surface quality even under a common slicing workflow.

Post-processing in the present workflow was intentionally limited in scope. Only strings and zits were removed, without mechanical finishing intended to alter layer lines or compensate for geometric deformation. Under this constraint, zits were removed slightly more easily on ABS, whereas stringing was removed more easily on PLA than on PETG. This observation is reported as a qualitative indicator of post-processing effort aligned with the dominant defect type and should not be interpreted as a quantitative comparison.

### 3.2. Slicer Mass Prediction Error

**Figure 3:**
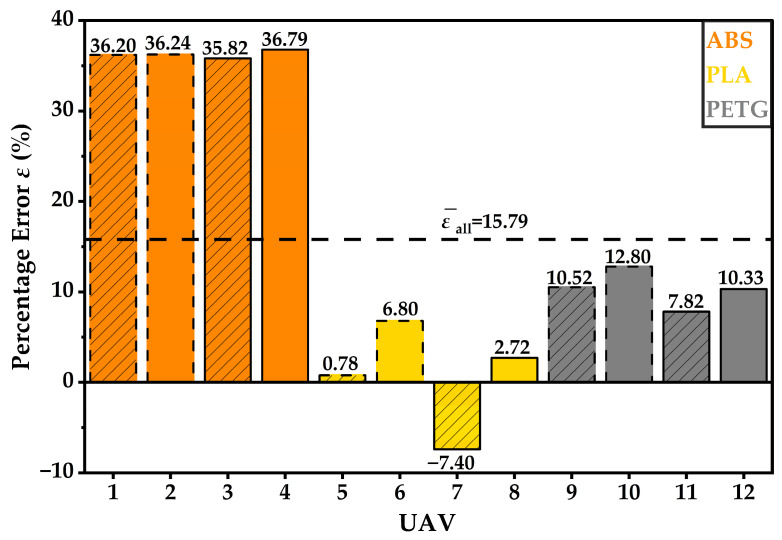
Percentage error *ε* (%) between m_slicer_ and m_real_ for the 12 specimens (UAV 1–12). The dashed line indicates the overall mean, ${\bar{\varepsilon }}_{\mathrm{all}}$.

Specimen-level percentage error *ε* is reported for all twelve prints in Figure 3, and the overall mean ${\bar{\varepsilon }}_{\mathrm{all}}$ is shown as a dashed reference line.

At specimen level, the three materials exhibit distinct error signatures. ABS values are tightly clustered near 36% for both designs and both infill levels, indicating a repeatable positive bias under the examined ABS workflow. PETG remains positive across all four prints but spans a wider range, from 7.82% to 12.80%, indicating systematic overestimation with moderate configuration sensitivity. PLA exhibits both negative and positive deviations, ranging from −7.40% to 6.80%, yielding a near-zero mean while producing the largest dispersion among the materials.

**Figure 4:**
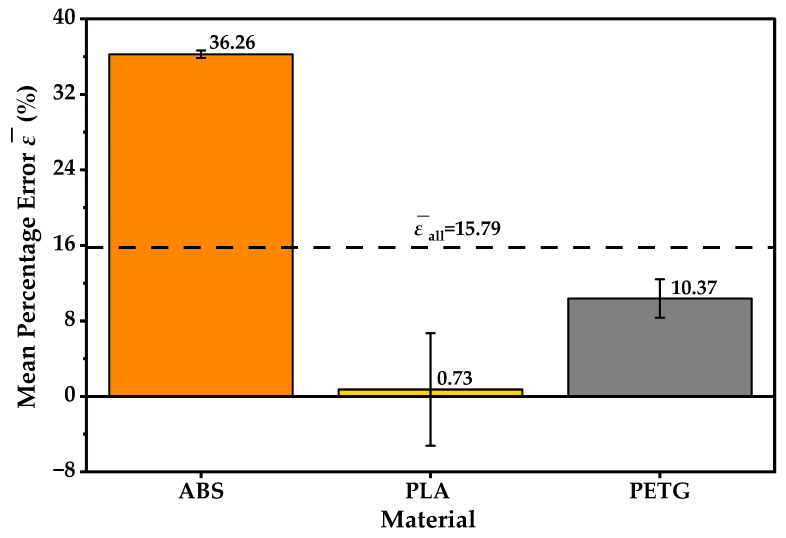
Mean percentage error $\bar{\varepsilon }$ (%) by material (n = 4). Error bars denote ±s*_ε_*, and the dashed line indicates the overall mean, ${\bar{\varepsilon }}_{\mathrm{all}}$.

Grouped results by material and by frame design are provided in Table 3 and Table 4 and are also visualised in Figure 4.

**Table 3:** Mean percentage mass error (%) by material (n = 4 per material).

Material	n (UAV)	$\mathrm{Mean}\;\bar{\varepsilon }$ (%)	s_ε_ (%)
ABS	4	36.26	0.40
PETG	4	10.37	2.04
PLA	4	0.73	5.97

**Table 4:** Mean percentage mass error (%) by frame design (n = 6 per design).

Design	n (UAV)	$\mathrm{Mean}\;\bar{\varepsilon }$ (%)	s_ε_ (%)
micro105	6	17.22	15.27
MK8	6	14.35	18.06

**Figure 5:**
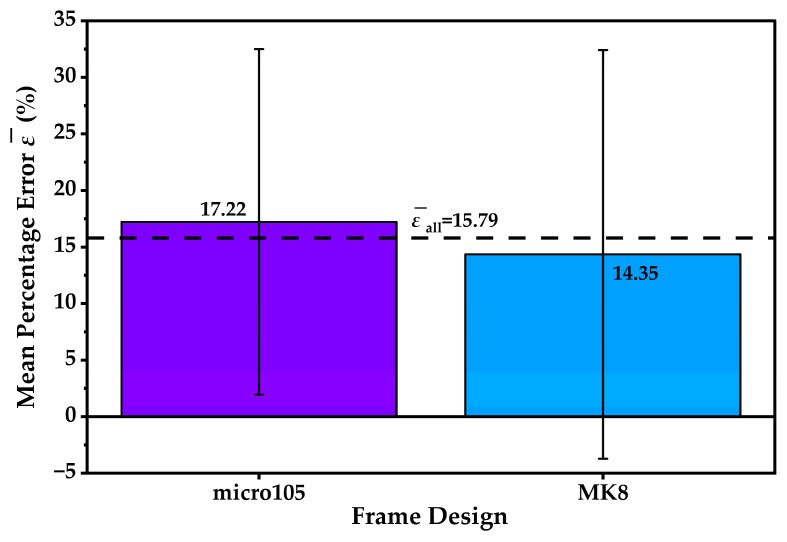
Mean percentage error $\bar{\varepsilon }$ (%) by frame design (n = 6 per design). Error bars denote ±s*_ε_*, and the dashed line indicates the overall mean, ${\bar{\varepsilon }}_{\mathrm{all}}$.

At the material level, slicer bias was strongly material-dependent within this dataset. ABS showed a consistent overestimation, with mean $\bar{\varepsilon }$ = 36.26% and s*_ε_* = 0.40%. PETG exhibited an intermediate overestimation, with $\bar{\varepsilon }=10.37\%$ and s*_ε_* = 2.04%. PLA remained close to zero mean bias with $\bar{\varepsilon }=0.73\%$, but exhibited the largest dispersion with s*_ε_* = 5.97%. The error bars in Figure 4 therefore convey two distinct features: the magnitude of systematic bias, which is highest for ABS and moderate for PETG, and configuration-dependent variability, which is most pronounced for PLA in the present sample. The mean percentage errors $\bar{\varepsilon }$ (%) by frame design are visualised in Figure 5.

When grouped by design, the micro105 group yielded $\bar{\varepsilon }$ = 17.22% with s*_ε_* = 15.27%, while MK8 yielded $\bar{\varepsilon }$ = 14.35% with s*_ε_* = 18.06%. The two design-level means are of similar magnitude relative to the large dispersions. This pattern is expected because each design group pools specimens from three materials that exhibit substantially different bias magnitudes. Accordingly, design-level summaries in the present dataset provide a secondary view of the results but do not support a material-neutral design effect on slicer mass prediction.

**Figure 6:**
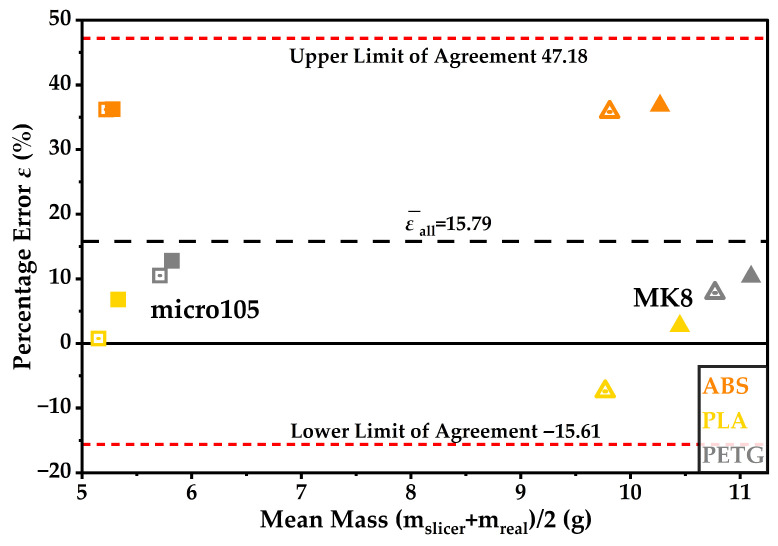
Bland–Altman plot (percentage basis) comparing m_slicer_ and m_real_. The central dashed line indicates ${\bar{\varepsilon }}_{\mathrm{all}}$, and the red dashed lines show the 95% limits of agreement.

Overall agreement between m_slicer_ and m_real_ is further examined using Bland–Altman analysis on a percentage basis in Figure 6.

The overall mean percentage error was ${\bar{\varepsilon }}_{\mathrm{all}}$ = 15.79%, with 95% limits of agreement from −15.61% to 47.18%. Expressing agreement on a percentage basis is appropriate here because it enables a direct comparison across lighter and heavier micro-frames without disproportionately weighting higher-mass specimens. Within the combined view, ABS points concentrate at high positive deviations, consistent with the high mean bias and low dispersion observed for ABS in the grouped material statistics. This behaviour is consistent with the systematic overestimation observed for ABS and can be attributed to the combined effects of material density assumptions, thermal shrinkage, and process-induced deviations from nominal deposition volume, as discussed further in Section 4. These limits should be interpreted as indicative given the modest sample size, but they provide a compact error envelope for slicer performance under conditions comparable to the present experimental set.

Finally, across the present specimen set, reducing infill from 100% to 35% corresponded to small absolute differences in measured mass, ranging from 0.02 g to 0.35 g. This range is reported here to contextualise the scale of the parts and the limited mass leverage of infill reduction at the micro-frame level under the present settings, where shells, perimeters, and locally solid regions around functional features contribute a substantial fraction of total mass.

### 3.3. Dimensional Consistency of Motor Mount Height

**Figure 7:**
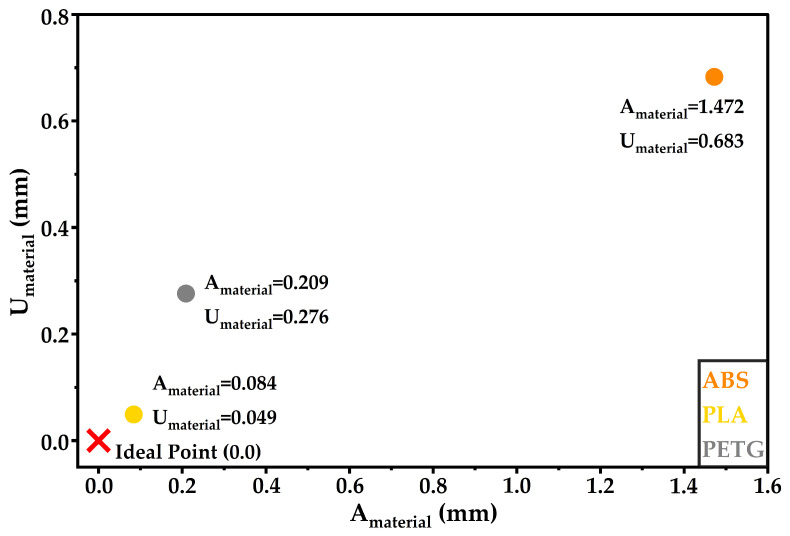
Summary of A_material_ and U_material_ (mm) for ABS, PETG, and PLA. The point (0.0) represents the ideal case of zero deviation from the nominal CAD geometry and perfect uniformity across the motor mount bases.

Dimensional consistency was evaluated at the motor mount bases, geometrically critical features for assembly and for maintaining relative alignment across the four motor locations. Dimensional deviations at motor mounts may lead to small thrust vector misalignments, which can affect flight stability in lightweight UAV platforms. Frame-level indices are reported for micro105 and MK8 in Table 5 and Table 6, respectively. Material-level indices aggregated across designs and infill levels are reported in Table 7 and summarised in Figure 7.

**Table 5:** A_UAV_ and U_UAV_ (mm) for the micro105 design (h_nom_ = 7.50 mm), by material and infill level.

Material	Infill (%)	A_UAV_ (mm)	U_UAV_ (mm)
ABS	35	1.972	1.491
ABS	100	0.145	0.175
PETG	35	0.084	0.031
PETG	100	0.296	0.407
PLA	35	0.127	0.056
PLA	100	0.135	0.058

**Table 6:** A_UAV_ and U_UAV_ (mm) for the MK8 design (h_nom_ = 10.00 mm), by material and infill level.

Material	Infill (%)	A_UAV_ (mm)	U_UAV_ (mm)
ABS	35	0.695	0.156
ABS	100	3.076	0.910
PETG	35	0.335	0.437
PETG	100	0.121	0.230
PLA	35	0.033	0.051
PLA	100	0.041	0.029

**Table 7:** Overall material-level dimensional indices.

Material	A_material_ (mm)	U_material_ (mm)
ABS	1.472	0.683
PETG	0.209	0.276
PLA	0.084	0.049

For the micro105 design (h_nom_ = 7.50 mm), PLA showed low deviations and stable uniformity at both infill levels, with A_UAV_ near 0.13 mm and U_UAV_ near 0.06 mm for both 35% and 100% infill. PETG yielded its lowest deviation and best uniformity at 35% infill (A_UAV_ = 0.084 mm and U_UAV_ = 0.031 mm), but performance degraded at 100% infill, particularly in uniformity (U_UAV_ = 0.407 mm). ABS’s performance was strongly related to the utilisation of the adhesion aid. At 35% infill, both deviation and non-uniformity were high (A_UAV_ = 1.972 mm and U_UAV_ = 1.491 mm), whereas at 100% infill, when the adhesion aid was employed, both indices decreased substantially (A_UAV_ = 0.145 mm and U_UAV_ = 0.175 mm).

For the MK8 design (h_nom_ = 10.00 mm), PLA again maintained very low deviation and good uniformity at both infill levels, with A_UAV_ between 0.033 mm and 0.041 mm and U_UAV_ between 0.029 mm and 0.051 mm. PETG exhibited elevated deviation and reduced uniformity at 35% infill (A_UAV_ = 0.335 mm and U_UAV_ = 0.437 mm), while 100% infill improved both indices (A_UAV_ = 0.121 mm and U_UAV_ = 0.230 mm). Again, ABS produced its most severe dimensional deviation in MK8 at 100% infill (A_UAV_ = 3.076 mm and U_UAV_ = 0.910 mm), where no adhesion aid was utilised. This configuration coincides with the pronounced warping observed in Figure 2a, consistent with the expectation that macroscopic deformation is accompanied by dimensional instability at the motor mounts.

At the material level, the aggregated indices in Table 5 and the scatter summary in Figure 7 identify PLA as the best overall performer in the present dataset, with low deviation from nominal and high inter-base uniformity (A_material_ = 0.084 mm and U_material_ = 0.049 mm). In this two-dimensional representation, points closer to the ideal point at (0.0) indicate more favourable dimensional performance, as they combine lower deviation from the nominal geometry with greater uniformity across the motor mount bases. PETG showed an intermediate overall dimensional performance with indices (A_material_ = 0.209 mm and U_material_ = 0.276 mm) and exhibited configuration sensitivity across designs and infill levels. ABS yielded the largest overall deviations (A_material_ = 1.472 mm and U_material_ = 0.683 mm), driven by the two micro-frames that were printed without any adhesion aid and exhibited pronounced warping.

Across the examined conditions, a clear material-dependent ranking emerged. PLA showed the most favourable overall performance, combining the highest dimensional fidelity with near-unbiased slicer mass prediction. PETG exhibited intermediate behaviour, with acceptable dimensional performance but greater configuration sensitivity. ABS showed the lowest dimensional stability, primarily due to warping-related distortion, and also exhibited the largest positive mass prediction bias. These combined findings indicate that, within the present process window, material selection is a dominant factor governing both dimensional accuracy and manufacturing predictability in FFF-printed UAV micro-frames.

## 4. Discussion

This study compared ABS, PETG, and PLA for the fabrication of UAV micro-frames by FFF across two representative geometries and two infill levels, using a common slicing workflow and consistent evaluation metrics. The evidence was synthesised along three axes: qualitative print-defect documentation, slicer mass prediction error expressed as percentage error *ε* between m_slicer_ and m_real_, and motor mount dimensional consistency quantified through frame-level indices and aggregated material-level indices. The present study focuses on manufacturing fidelity, defect manifestation, and slicer mass prediction reliability for FFF-fabricated micro-frames. Mechanical characterisation of the printed structures was not included in the present experimental protocol. Future work could extend the present analysis through tensile, flexural, or impact testing in order to relate the observed dimensional fidelity and defect formation to the mechanical performance of UAV structural components. From an application perspective, these findings indicate that even small dimensional deviations at motor mount features can translate into assembly misalignment and potential performance degradation in UAV systems, highlighting the importance of process-aware material selection.

The defect inspection differentiates ABS from the other two materials under the present process window. Importantly, these defects are not only visual artefacts but are directly linked to dimensional performance. Warping in ABS corresponds to large geometric deviations and reduced dimensional consistency, while surface defects such as stringing and zits in PLA and PETG have a more limited impact on critical dimensional features but may still affect local tolerances and surface quality relevant to assembly. ABS exhibited pronounced warping and zits, consistent with an elevated risk of gross deformation at the micro-frame scale. In contrast, PLA and PETG showed no macroscopic warping in this dataset, and their dominant defects were surface-related, primarily stringing, zits, and layer lines. The grading also indicates that surface quality is not purely material driven. For PETG, layer lines were more pronounced in MK8 than in micro105, suggesting that geometry and toolpath interactions can remain visible even when slicing is kept consistent.

The pronounced warping observed in ABS can be interpreted in the context of its thermomechanical behaviour during cooling. ABS is an amorphous polymer with a relatively high glass transition temperature (Tg ≈ 105 °C), which results in a prolonged transition from a viscoelastic to a rigid state during layer solidification. During this transition, thermal gradients develop between newly deposited material and previously solidified layers, leading to the accumulation of residual stresses. These stresses are further amplified by differential cooling across the geometry, particularly in regions with varying thickness such as motor mount features. Although enclosed printing conditions were employed to mitigate rapid heat loss, complete thermal homogenization is not achieved, and localised shrinkage effects persist. In contrast, PLA, with a lower Tg and reduced thermal contraction, undergoes a more stable solidification process, resulting in improved dimensional fidelity and reduced susceptibility to warping.

These observations are consistent with previous studies reporting increased warping and dimensional instability in ABS compared to PLA and PETG under comparable FFF conditions, as well as improved dimensional stability in PLA due to its lower thermal shrinkage. The present results extend these findings by demonstrating their impact on complex, small-scale geometries with functionally critical features, such as UAV micro-frames, where dimensional deviations directly affect assembly quality.

A second robust outcome concerns slicer mass prediction. The results demonstrate a strongly material-dependent bias pattern. ABS showed a large and highly consistent positive bias, with mean $\bar{\varepsilon }$ = 36.26% and low dispersion. PETG exhibited an intermediate positive bias with $\bar{\varepsilon }$ = 10.37% and PLA remained near-unbiased on average, with $\bar{\varepsilon }$ = 0.73%, but displayed the largest dispersion, consistent with specimen-level deviations that changed sign. When the data are grouped by design, the resulting means are similar in magnitude relative to large dispersions. This is expected because each design group pools materials with substantially different *ε* signatures. The combined agreement envelope across all specimens is wide, with ${\bar{\varepsilon }}_{\mathrm{all}}$ = 15.79% and 95% limits of agreement from −15.61% to 47.18%. Under comparable conditions, slicer-based mass estimates should not be treated as interchangeable with measured mass without material-specific validation. Importantly, this mass prediction error is not a material quality criterion. It reflects slicer bias and highlights the need for material-specific calibration, rather than intrinsic polymer superiority or inferiority.

At the examined micro-frame scale, reducing infill from 100% to 35% did not provide an appreciable mass reduction. Across the specimen set, the measured mass difference associated with infill reduction ranged from 0.02 g to 0.35 g. Within this range and scale, infill selection should not be justified primarily as a strategy for mass reduction. If 35% honeycomb infill is selected, the motivation should be anchored in objectives other than mass, such as print time or structural performance considerations, which were not directly quantified in the present work.

The observed discrepancies between slicer-predicted and experimentally measured mass can be attributed to several interacting factors related to both material properties and process calibration. Slicer software (in our case Simplify3D v4.1.2) typically estimates part of the mass based on nominal filament density values provided by the user or manufacturer; however, deviations between nominal and actual material density can introduce systematic bias. In addition, process-induced porosity and localised under-extrusion may reduce the effective deposited volume, particularly in materials with higher melt viscosity or less stable flow behaviour. Thermal shrinkage also plays a role, as materials such as ABS exhibit greater volumetric contraction during cooling, leading to a reduced final part volume compared to the nominal CAD geometry assumed by the slicer. Finally, differences in extrusion multiplier and flow calibration between materials and printer configurations can further affect the actual mass of deposited material. These combined effects provide a plausible explanation for the material-dependent bias observed in slicer mass prediction, with ABS showing the largest deviation, PETG intermediate behaviour, and PLA near-unbiased performance under the present conditions.

Dimensional consistency at the motor mount bases further differentiates the materials. At the material level, PLA achieved the most favourable overall dimensional performance, with A_material_ = 0.084 mm and U_material_ = 0.049 mm. PETG was intermediate, with A_material_ = 0.209 mm and U_material_ = 0.276 mm, and exhibited configuration sensitivity. ABS exhibited the least favourable overall dimensional behaviour, with A_material_ = 1.472 mm and U_material_ = 0.683 mm. The most severe dimensional deviations occurred for ABS in the two specimens where no adhesion aid was employed. Collectively, the dimensional indices support evidence-based process direction for UAV micro-frames.

The material-dependent behaviour observed in this study can be further interpreted in terms of fundamental polymer processing characteristics. Melt rheology plays a critical role in deposition stability, with materials exhibiting higher viscosity or less stable flow behaviour being more susceptible to irregular extrusion and defect formation. Interlayer adhesion is governed by the degree of polymer chain diffusion across deposited filaments, which depends on the temperature, viscosity, and cooling rate. From a structural standpoint, ABS is an amorphous polymer, whereas PLA and PETG exhibit semi-crystalline or partially crystalline characteristics, influencing their shrinkage behaviour and thermal response. Amorphous polymers such as ABS tend to exhibit higher and more isotropic thermal contraction, increasing their sensitivity to cooling gradients and warping. In contrast, PLA, with lower thermal shrinkage and more stable solidification behaviour, demonstrates improved dimensional fidelity. PETG exhibits intermediate behaviour, combining relatively good layer adhesion with moderate sensitivity to cooling conditions. These combined rheological, structural, and thermomechanical factors provide a coherent explanation for the differences in defect manifestation and dimensional performance observed across the examined materials.

The findings should be interpreted within the experimental scope of the present study, which includes specific process conditions and environmental factors that may influence material behaviour. The investigation focused on a controlled factorial comparison of three widely used thermoplastic materials across two representative UAV micro-frame geometries and two infill levels under a common slicing workflow. The adopted setup reflects a realistic benchtop additive manufacturing environment in which printer configuration, material choice, and process parameters interact in practice. Within this defined framework, the results provide comparative insight into material-dependent defect behaviour, slicer mass prediction bias, and dimensional fidelity at functionally critical motor mount features. In particular, environmental conditions such as ambient temperature and humidity, as well as printer-specific thermal stability, may affect warping behaviour and dimensional accuracy, especially for materials such as ABS.

## 5. Conclusions

This study examined the fabrication of UAV micro-frames by fused filament fabrication using ABS, PETG, and PLA, focusing on dimensional fidelity, defect manifestation, and slicer mass prediction accuracy.

From a practical standpoint, PLA exhibited the most stable dimensional performance and the lowest overall deviation from the nominal CAD geometry, making it the most suitable material for UAV micro-frame fabrication within the present process window. PETG demonstrated intermediate behaviour with configuration-dependent variability and may be considered a viable alternative where increased ductility is required, while ABS presented the most severe dimensional distortions associated with warping under the examined conditions.

Slicer-based mass estimation showed a strong material-dependent bias. ABS builds exhibited a large and consistent overestimation, PETG showed moderate overestimation, and PLA remained near-unbiased on average but with higher dispersion.

At the micro-frame scale examined here, reducing the infill density from 100% to 35% produced only minor reductions in the measured mass. Under comparable conditions, infill selection should therefore be motivated primarily by objectives such as print time or structural performance rather than mass reduction alone. More broadly, these findings highlight that material-dependent behaviour in FFF should be carefully considered when dimensional accuracy and process predictability are critical, extending beyond UAV applications to other small-scale functional components.

Overall, the results provide practical guidance for material selection and process calibration when dimensional fidelity and manufacturing reliability are critical for small-scale FFF polymer components. Future work should focus on the mechanical characterisation of the printed components and the optimisation of process parameters, particularly for materials such as ABS, where improved thermal management may enhance performance.

## Figures and Tables

**Table 1:** Print parameters per material.

Parameter	ABS	PETG	PLA
Print speed (mm/s)	45	45	45
Nozzle diameter (mm)	0.4	0.4	0.4
Extrusion width (%)	100	100	100
Nozzle temperature (°C)	250	230	210
Bed temperature (°C)	90	60	60
Extrusion multiplier (%)	100	96	90
Retraction speed (mm/s)	30	60	30
Retraction distance (mm)	1.0	8.5	5.0
**Special first-layer settings**			
Height (% of subsequent layers)	90	90	90
Print speed (mm/s)	22.5	36	36

**Table 2:** Concise qualitative assessment of print defects by material.

Material	Stringing	Zits	Layer Lines	Warping
ABS	none	pronounced	none	pronounced
PETG	moderate	moderate	moderate (micro105) pronounced (MK8)	none
PLA	pronounced	slight	slight	none

## Data Availability

The original contributions presented in this study are included in the article. Further inquiries can be directed to the corresponding author.

## Abbreviations

The following abbreviations are used in this manuscript:

ABS	Acrylonitrile–Butadiene–Styrene
AM	Additive Manufacturing
CAD	Computer-Aided Design
FDM	Fused Deposition Modelling
FFF	Fused Filament Fabrication
LoA	Limit of Agreement
MEX	Material Extrusion
PETG	Polyethylene Terephthalate Glycol
PLA	Polylactic Acid
STL	Stereolithography
UAV	Unmanned Aerial Vehicle
